# Supplementary material for: Chemical Composition and Biological Activities of Essential Oils from Origanum vulgare Genotypes Belonging to the Carvacrol and Thymol Chemotypes
Source: Plants (Basel). 2023 Mar 16;12(6):1344. doi: 10.3390/plants12061344 (PMC10059975; doi:10.3390/plants12061344)
Supplement: Supplementary file 1 [file plants-12-01344-s001.zip › plants-2272079-supplementary.pdf]

Supplementary data

Table S1. List of quantified constituents of oregano essential oils. Retention indexes and method of identification used.

Nr	Compound name	CAS Registry number	Class	Retention index experimental	Retention index, from standard pure compounds or literature[1]	Method of identification[2]
1	α-thujene	3917-48-4	MH[3]	925	916-938	RI, MS
2	α -pinene	80-56-8	MH	933	934	RI, MS, PC
3	1-octen-3-ol	3391-86-4	Ot	961	961	RI, MS, PC
4	3-octanone	106-68-3	Ot	964	965	RI, MS, PC
5	sabinene	3387-41-5	MH	968	958-981	RI, MS
6	β-pinene	127-91-3	MH	974	974	RI, MS, PC
7	β-myrcene	123-35-3	MH	982	982	RI, MS, PC
8	α-phellandrene	99-83-2	MH	1000	1001	RI, MS, PC
9	α-terpinene	99-86-5	MH	1011	1011	RI, MS, PC
10	*p*-cymene	99-87-6	MH	1014	1015	RI, MS, PC
11	limonene	138-86-3	MH	1023	1022	RI, MS, PC
12	*cis*-β-ocimene	3338-55-4	MH	1026	1017-1040	RI, MS
13	*tr*-β-ocimene	3779-61-1	MH	1037	1027-1050	RI, MS
14	γ-terpinene	99-85-4	MH	1050	1051	RI, MS, PC
15	*cis*-sabinene hydrate	15537-55-0	OM	1055	1044-1066	RI, MS
16	terpinolene	586-62-9	MH	1081	1081	RI, MS, PC
17	linalool	78-70-6	OM	1084	1085	RI, MS, PC
18	borneol	507-70-0	OM	1152	1151	RI, MS, PC
19	4-terpineol	562-74-3	OM	1165	1165	RI, MS, PC
20	thymol methyl ether	1076-56-8	OM	1215	1199-1235	RI, MS
21	carvacrol methyl ether	6379-73-3	OM	1225	1205-1230	RI, MS
22	thymol	89-83-8	OM	1267	1268	RI, MS, PC
23	carvacrol	499-75-2	OM	1282	1282	RI, MS, PC
24	β-caryophyllene	87-44-5	SH	1423	1423	RI, MS, PC

25	α-humulene	6753-98-6	SH	1456	1457	RI, MS, PC
26	γ-muurolene	30021-74-0	SH	1474	1455-1494	RI, MS
27	germacrene D	23986-74-5	SH	1481	1458-1491	RI, MS
28	bicyclogermacrene	67650-90-2	SH	1496	1474-1501	RI, MS
29	β-bisabolene	495-61-4	SH	1503	1485-1511	RI, MS
30	γ-cadinene	39029-41-9	SH	1511	1490-1521	RI, MS
31	δ-cadinene	483-76-1	SH	1518	1498-1526	RI, MS
32	unknown		SH	1641	-	-

[1] Reference retention indices were reported as a single value when they were determined on pure standard compounds or as a range when pure compounds were not available and data were retrieved from the literature. The reported range are the 90% confidence interval estimates of retention indices, based on the number of available data records, determined on dimethylsilicone stationary phase, as reported by Babushok et al., 2011.
[2] Identification of compound based on: RI (literature retention index), MS (library mass spectrum), PC (retention index and mass spectrum of pure standard compound).
[3] MH: Monoterpene Hydrocarbons; OM: Oxygenated Monoterpene; SH: Sesquiterpene Hydrocarbons; Ot: Others.

Table S2. Target genes and primer sequences.

GENE NAME	FORWARD PRIMER	REVERSE PRIMER
**IKBA**	CCTGACCTGGTGTCACTCCTG	TGCTGTATCCGGGTGCTTG
**CIAP2**	TGTTTCAGATCTACCAGTGG	GACACTTCTTTGTCCATACAC
**IL-6**	GTCAGGGGTGGTTATTGCAT	AGTGAGGAACAAGCCAGAGC
**IL-8**	GACATACTCCAAACCTTTCC	TTTATGAATTCTCAGCCCTC
**IL-1A**	CCGTGAGTTTCCCAGAAGAA	ACTGCCCAAGATGAAGACCA
**GAPDH**	GAAGAAATGCGAGATCCCT	ACTTCTCATGGTTCACACC
